# A Multidimensional Analysis of the Determinants of Medication Counseling Behaviors: A Cross-Sectional Study of Community Pharmacists in Jazan, Saudi Arabia

**DOI:** 10.7759/cureus.104708

**Published:** 2026-03-05

**Authors:** Raghid T Alharbi, Roaa H Alhazmi, Shahad M Alotaibi, Lojain A Adawi, Mosa J Alfaify, Amal A Jali, Riyadh T Izzuldeen, Sharifah J Alraythi, Khalid H Someli, Khawlah J Jerebi, Amirah M Hakami, Shouq B Algethami, Maryam I Shawk, Hassan A Madkhali, Ibrahim M Gosadi

**Affiliations:** 1 College of Pharmacy, Jazan University, Jazan, SAU; 2 Pharmacy, Moaz Pharma, Jazan, SAU; 3 College of Pharmacy, Shaqra University, Shaqra, SAU; 4 College of Pharmacy, Taif University, Taif, SAU; 5 Department of Family and Community Medicine, Jazan University, Jazan, SAU

**Keywords:** community pharmacy services, medication counseling, patient safety, pharmacy workforce, professional practice, saudi arabia

## Abstract

Background: Community pharmacists serve as the final safety checkpoint in the medication-use system; however, practice patterns often prioritize technical logistics over clinical safety. While the deficit in risk counseling is well-documented, the mechanisms driving this "selective negligence" remain underexplored.

Objective: To evaluate the structural and professional determinants of routine versus risk-based counseling behaviors among community pharmacists in Jazan, Saudi Arabia.

Methods: A cross-sectional analytical study was conducted (July-September 2024) among licensed community pharmacists (N=230) across the Jazan region. Data were collected using a multi-domain instrument assessing self-reported counseling frequencies. Multivariable beta regression models were employed to estimate the average marginal effects (AME) of predictors, adjusting for workforce structure, workload, and pharmacist demographics.

Results: Analysis revealed a functional dichotomy: "core use" instructions were universal (median: 5 (IQR: 5-5)), whereas "risk communication" was significantly lower (median: 4 (IQR: 2-5)). Regression models identified divergent determinants. "core use" was driven by professional habits; "checking patient understanding" was the strongest positive predictor (AME: +10.3%, p<0.001), while lower self-rated quality of counseling significantly reduced performance (AME: -10.7%, p<0.001). Conversely, "risk communication" was structurally driven; the presence of a pharmacy assistant was the primary positive predictor (AME: +9.9%, p=0.010). Notably, holding a Doctor of Pharmacy (PharmD) degree was associated with lower "core use" scores (AME: -4.7%, p=0.004) compared to BSc (Bachelor of Science in Pharmacy) degrees and showed no significant advantage for risk counseling.

Conclusion: Routine counseling is sustained by professional habit, but safety-critical risk communication is constrained by operational bandwidth. Closing the safety gap requires structural interventions, specifically the integration of support staff, rather than reliance on educational credentials alone.

## Introduction

Community pharmacies operate as the most accessible entry point to the primary healthcare system. They offer extended operating hours and allow patients to seek care without an appointment, features that position them uniquely to address immediate health needs [1-3]. International health organizations increasingly rely on pharmacists to act as the final safety checkpoint in the medication-use process. The International Pharmaceutical Federation (FIP) guidelines emphasize that dispensing is not merely a logistical transaction but a clinical encounter where the pharmacist must ensure the patient understands both how to use the medicine and the risks associated with it [4].

In Saudi Arabia, the sector has expanded rapidly to meet these expectations. Recent data indicate the pharmacy network has grown substantially, with the Jazan region reporting a density of approximately 3.91 pharmacies per 10,000 inhabitants [5,6]. Despite this physical availability, the functional integration of clinical services remains limited. Studies suggest that public interaction with community pharmacies in the Kingdom remains predominantly transactional [7-9]. Patients often view the pharmacist’s role as confined to product supply rather than cognitive service provision, a perception that may stem from inconsistent counseling practices at the counter.

Current evidence reveals that when counseling does occur, it often follows a pattern of selective omission. Pharmacists frequently prioritize routine technical instructions, such as dosage and administration routes, while neglecting complex safety information [10-13]. This imbalance is critical because communicating risk differs fundamentally from providing instructions. Ferner and Aronson (2006) argue that explaining potential harm involves a sophisticated negotiation of benefit versus risk that requires higher cognitive engagement than standard directive counseling [14]. Consequently, a pharmacist might consistently provide excellent instruction on how to take a drug while simultaneously failing to check whether the patient understands why they should be cautious.

Determining why these disparities exist requires analyzing the tension between the pharmacist’s internal capacity and their external work environment. On one hand, individual attributes such as self-efficacy and professional commitment are strong predictors of performance. Yorimoto et al. (2022) demonstrated that pharmacists who possess high confidence in their abilities are more likely to engage in comprehensive counseling behaviors [15]. Similarly, Noureldin and Melton (2020) link self-efficacy directly to the successful implementation of patient-care processes. These findings suggest that the drive to counsel is partly internal [16].

On the other hand, the practice environment imposes structural constraints that can suppress these internal drivers. High prescription volumes and administrative burdens compete for the pharmacist’s limited time and cognitive bandwidth [17]. Emerging literature highlights the role of support staff in mitigating this pressure. Walker et al. (2020) found that effective delegation of technical tasks to pharmacy technicians significantly increased the time pharmacists spent on direct patient care [18]. By offloading logistical duties, the practice environment can theoretically create the mental space required for complex risk communication [19].

Research often aggregates counseling into a single quality score, potentially obscuring how these distinct factors influence specific behaviors. It remains unclear whether the factors that drive routine instruction are the same as those that enable safety counseling. This study addresses this knowledge gap by examining the determinants of point-of-sale (POS) counseling among community pharmacists in Jazan. We aim to assess self-reported counseling frequencies and identify how pharmacist-level factors, such as self-rated quality, and practice-level factors, such as the presence of assistants, differentially associate with the provision of core use instructions versus risk communication.

## Materials and methods

Study design and participants

A cross-sectional survey of community pharmacists in the Jazan region of Saudi Arabia was conducted between July 1 and September 1, 2024. The study setting included community pharmacies across the region, where the workforce comprises two distinct cadres: licensed pharmacists (holding a BSc (Bachelor of Science in Pharmacy) or Doctor of Pharmacy (PharmD) degree) who have full clinical and dispensing privileges, and pharmacy assistants (typically holding a diploma). In this context, assistants primarily manage logistical tasks, such as inventory control, billing, and packaging, but are not licensed to provide clinical counseling.

Participants were recruited using a non-probability convenience sampling strategy, selecting one licensed pharmacist with at least two years of experience per pharmacy to maximize geographic dispersion. The target sample size was calculated as 227 (based on a population of 549 pharmacies, 95% confidence level, and 5% margin of error) [6]. Of 273 pharmacists approached, 230 provided complete data, satisfying the power requirements.

Data collection instrument

Data were collected using a multi-domain questionnaire designed to capture counseling behaviors and their potential determinants. The primary outcome was measured using an eight-item composite index of counseling frequency, adapted from FIP guidelines [4]. This index specifically asked pharmacists to report the frequency of providing eight distinct types of medication information (e.g., dose, side effects, contraindications) on a five-point ordinal scale (1=never to 5=always).

The questionnaire also assessed three categories of determinants: (a) professional perceptions, including self-rated counseling quality (excellent vs. good/fair) and the frequency of checking patient understanding (frequent vs. less frequent); (b) practice environment, specifically the presence of a pharmacy assistant (yes/no) and workload pressure; and (c) demographics, including age, academic degree, and daily patient load. The full instrument is provided in Appendix 1.

Structural assessment of counseling domains

To determine whether counseling is a unidimensional behavior or comprises distinct domains, we performed an exploratory factor analysis (EFA) on the eight-item inventory. We assessed the factor structure using maximum likelihood estimation with an oblimin rotation to account for potential correlations between factors. The number of factors to retain was determined using a scree plot inspection and parallel analysis. Internal consistency for the resulting subscales was evaluated using Cronbach’s α and McDonald’s ωₜ. For two-item subscales, the Spearman-Brown coefficient was calculated as the most appropriate reliability metric.

Data analysis

Counseling scores were normalized to the open interval (0,1) using the Smithson-Verkuilen transformation. Because the outcome data were bounded and exhibited a skewness characteristic of self-reported performance, ordinary least squares (OLS) regression assumptions were violated. Therefore, beta regression with a logit link was selected as the appropriate method for modeling rates and proportions [20,21].

Three separate multivariable beta regression models were specified to predict the overall, core use, and risk communication scores. Models were adjusted for pharmacist age, degree, experience, and patient load. Prior to finalizing the models, potential multicollinearity among predictor variables was evaluated using variance inflation factors (VIF), confirming the absence of any significant collinearity. Further, to provide clinically intuitive interpretations, results are reported as average marginal effects (AME). The AME represents the estimated percentage-point change in the counseling score associated with a specific predictor, holding all other variables constant. All analyses were conducted using R version 4.4.0 [22]. Model fit was assessed using the Akaike information criterion (AIC).

Ethical approval

The study adhered to the Declaration of Helsinki and received ethical approval from the Standing Committee for Scientific Research at Jazan University, KSA (Reference Number: REC42/1/142; HAPO-10-Z-001).

## Results

Structural validity and psychometric properties

**Figure 1:**
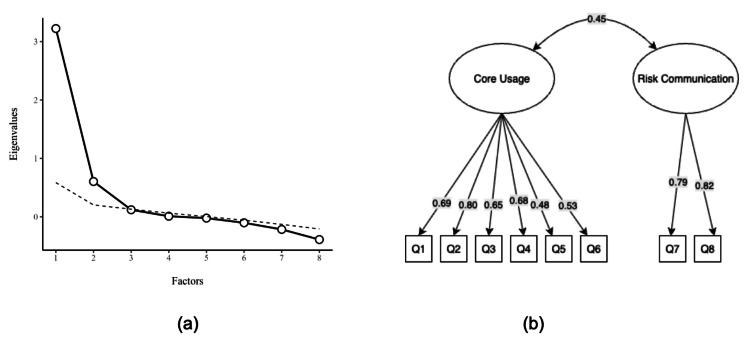
Factor structure of the CCI-8 derived from EFA The figure displays the results of the EFA conducted on the eight-item CCI. (a) The scree plot graphs the eigenvalues for each potential factor; the point of inflection after the second factor supported a two-factor solution, a decision corroborated by parallel analysis. (b) The path diagram illustrates the final two-factor solution derived using maximum likelihood estimation with an oblimin rotation. Ovals represent the latent factors ("core-use" and "risk-communication"), while rectangles represent the observed items (Q1-Q8). Values on the straight arrows are the standardized factor loadings from the pattern matrix, and the value on the curved, double-headed arrow represents the correlation between the two factors. EFA, exploratory factor analysis; CCI, Counseling Comprehensiveness Index

The internal structure of the counseling inventory was evaluated to determine whether practice patterns reflected a single behavior or distinct domains. The EFA supported a two-factor solution, which cumulatively explained 53.0% of the total variance. As illustrated in the scree plot (Figure 1a), the eigenvalues exhibited a sharp point of inflection after the second factor, providing empirical justification for retaining two distinct dimensions rather than a unidimensional structure. This separation is visualized in the path diagram (Figure 1b), which confirms a "simple structure" where routine technical items cluster onto a primary latent variable ("core use"), while safety warnings cluster onto a secondary, distinct variable ("risk communication"). The moderate correlation between these factors (r=0.45) suggests that while these behaviors are related, they function as independent constructs in clinical practice.

The specific composition of these factors is detailed in Table 1. The "core use" domain comprised six items related to logistical instructions (e.g., dose, route, and directions), with factor loadings ranging from 0.35 to 0.61. In contrast, the "risk communication" domain was defined exclusively by the two safety-critical items: "side effects" and "contraindications," which showed strong loadings of 0.59 and 0.60, respectively.

**Table 1:** Item-level descriptive statistics and factor loadings derived from EFA Extraction method: maximum likelihood with oblimin rotation. Factor loadings presented are from the Schmid-Leiman bifactor solution. Model fit indices: χ²(13)=28.58, p=0.008; RMSEA=0.072 (90% CI: 0.036, 0.108); BIC=-42.12. The correlation between the "core-use" and "risk-communication" factors was r=0.45. EFA, exploratory factor analysis; BIC, Bayesian information criterion; RMSEA, root mean square error of approximation

Item	Subscale	Mean (SD)	h²	Item-total correlation	Factor loading
Indication	Core use	4.50 (0.90)	0.43	0.57	0.51
Route of administration	Core use	4.61 (0.86)	0.58	0.62	0.61
Dose	Core use	4.70 (0.69)	0.44	0.59	0.48
Directions for use	Core use	4.52 (0.87)	0.57	0.68	0.51
Duration of therapy	Core use	4.51 (0.85)	0.43	0.57	0.35
Special directions	Core use	4.16 (1.09)	0.46	0.61	0.40
Side effects	Risk communication	3.48 (1.23)	0.67	0.67	0.59
Contraindications	Risk communication	3.73 (1.26)	0.63	0.67	0.60

Reliability testing, presented in Table 2, confirmed that this structural division yielded internally consistent scales. The overall eight-item index demonstrated high reliability (Cronbach’s α=0.84), as did the "core use" subscale (α=0.83). The two-item "risk communication" subscale also demonstrated acceptable reliability for a short scale (Spearman-Brown coefficient=0.80), supporting its use as an independent outcome measure in the subsequent analyses.

**Table 2:** Psychometric properties and reliability statistics for the counseling index and subdomains *For the two-item "risk-communication" subscale, the Spearman-Brown coefficient (0.80) is reported, which is equivalent to Cronbach's α for two-item scales. McDonald's ωₜ is not computed for two-item factors. α, Cronbach’s alpha; ωₜ, McDonald’s omega total

Scale/subscale	No. of items	Mean (SD)	α	ωₜ
Overall CCI-8 scale	8	4.25 (0.73)	0.84	0.87
Factor 1: core use	6	4.50 (0.71)	0.83	0.84
Factor 2: risk communication	2	3.61 (1.15)	0.80^*^	N/A

Participant demographics and practice characteristics

The study sample comprised 230 community pharmacists, representing a predominantly early-career workforce (Table 3). Nearly half of the participants, 112 (48.7%), were aged 20-30 years, and the sample was evenly distributed between those holding a PharmD degree, 115 (50.0%), and those with a BSc, 106 (46.1%). A notable structural dichotomy was observed in the practice environment: while the majority, 147 (63.9%), practiced with the support of a pharmacy assistant, a significant minority, 83 (36.1%), managed the dispensing workflow without support staff. The median daily patient load was 51 prescriptions (IQR: 31-61).

**Table 3:** Demographic characteristics and practice environment of community pharmacists (n=230) Data are presented as frequency (%) for categorical variables and median (IQR) for continuous variables. BSc, Bachelor of Science in Pharmacy; PharmD, Doctor of Pharmacy

Characteristics	N (%)
Pharmacist demographics	
Age group, n (%)	
20-30 years old	112 (49%)
31-40 years old	100 (43%)
41-50 years old	12 (5.2%)
>50 years old	6 (2.6%)
Academic degree, n (%)	
BSc	106 (46%)
PharmD	115 (50%)
Technician pharmacists	9 (4%)
Years in practice, n (%)	
<5 years	88 (38%)
5-10 years	93 (40%)
10-15 years	31 (13%)
15+ years	18 (8%)
Practice environment	
Pharmacy has assistant, n (%)	
No	83 (36%)
Yes	147 (64%)
Total patient load (per day), median (Q1, Q3)	51 (31, 61)

Frequency of counseling behaviors

A distinct discordance was observed between the provision of routine technical instructions and safety-related information (Table 4). Pharmacists reported near-universal adherence to directive counseling; the median frequency for discussing "dose," "route of administration," and "directions for use" was the maximum score of five (IQR: 5-5). In contrast, engagement in risk communication was significantly lower, with "side effects" and "contraindications" yielding a median frequency of four (IQR: 2-5) and four (IQR: 3-5), respectively.

**Table 4:** Frequency of self-reported POS counseling behaviors Responses were recorded on a five-point ordinal frequency scale ranging from 1 (never) to 5 (always). POS, point-of-sale

Self-reported counseling practices	Median (IQR)
Indications	5 (4, 5)
Route of administration	5 (5, 5)
Dose	5 (5, 5)
Directions for use	5 (4, 5)
Duration of therapy	5 (4, 5)
Special directions	5 (4, 5)
Side effects	4 (2, 5)
Contraindications	4 (3, 5)

Pharmacist perceptions, knowledge, and barriers

Despite the observed gaps in safety counseling, participants demonstrated robust professional self-efficacy (Table 5). The vast majority of respondents, specifically 177 (76.9%), rated their knowledge of drug-related problems (DRPs) as either "knowledgeable" or "very knowledgeable," and 200 (87.0%) rated their overall counseling quality as "good" or "excellent." However, this high internal confidence was juxtaposed against significant environmental constraints. The most commonly cited barrier to patient counseling was "workload pressure," reported by 138 (60.0%) respondents. This was followed by "lack of time," which was reported by 105 (45.7%) respondents. Consequently, the critical safety behavior of verifying patient understanding was performed inconsistently; fewer than half of the respondents, 114 (49.6%), reported "always" checking whether the patient understood the instructions.

**Table 5:** Professional perceptions, self-efficacy, and structural barriers to counseling DRPs, drug-related problems

Variable	n (%)
Checking patient understanding	
Never	1 (0.4%)
Sometimes	16 (7.0%)
Often	20 (8.7%)
Usually	79 (34.3%)
Always	114 (49.6%)
Overall rating of current counseling quality	
Excellent	68 (29.6%)
Good	132 (57.4%)
Fair	26 (11.3%)
Needs improvement	2 (0.9%)
Poor	2 (0.9%)
Agreement: consultations reduce DRPs	
Strongly agree	120 (52.2%)
Agree	74 (32.2%)
Slightly agree	29 (12.6%)
Slightly disagree	5 (2.2%)
Disagree	1 (0.4%)
Strongly disagree	1 (0.4%)
Perceived knowledge on DRPs	
Very knowledgeable	78 (33.9%)
Knowledgeable	99 (43.0%)
Moderately knowledgeable	49 (21.3%)
Unknowledgable	2 (0.9%)
Don't know	2 (0.9%)
Reported barriers to counseling	
Workload pressure	138 (60.0%)
Lack of time	105 (45.7%)
No patient history	88 (38.3%)
Lack of patient acceptance	86 (37.4%)
Lack of trust in pharmacist	56 (24.3%)
Lack of financial support	34 (14.8%)

Multivariable determinants of counseling domains

To identify the specific drivers of counseling behavior, we conducted multivariable beta regression analyses for the overall index and its two subdomains. The determinants of the overall Counseling Comprehensiveness Index (CCI) are presented in Table 6. This global model indicates that counseling performance is significantly influenced by professional habits; pharmacists who reported "frequently" checking patient understanding achieved scores 13.7 percentage points higher (AME=0.137, p<0.001) than those who checked less frequently. Experience also played a role, with senior pharmacists (>15 years of practice) showing a 10.2 percentage point increase (AME=0.102, p=0.013) over early-career peers. However, holding a PharmD degree was associated with an 8.6 percentage point decrease (AME=-0.086, p<0.001) in overall counseling scores compared to holding a BSc degree.

**Table 6:** Multivariable beta regression analysis of factors associated with the overall CCI score Model pseudo R²=0.230; AIC=-334.1, log likelihood=179. CCI, Counseling Comprehensiveness Index; AME, average marginal effect; SE, standard error; BSc, Bachelor of Science in Pharmacy; PharmD, Doctor of Pharmacy; AIC, Akaike information criterion

Predictor	AME (SE)	z-value	95% CI	p-value
Self-rated quality (vs. excellent)				
Good	-0.064 (0.020)	-3.17	(-0.104, -0.025)	0.002
Fair or lower	-0.141 (0.037)	-3.83	(-0.212, -0.069)	<0.001
Check understanding (vs. less frequent)				
Frequent	0.137 (0.033)	4.20	(0.073, 0.200)	<0.001
Barrier: workload pressure (vs. no)	0.034 (0.021)	1.64	(-0.007, 0.074)	0.101
Pharmacy has assistant (vs. no)	0.054 (0.022)	2.42	(0.010, 0.098)	0.016
Adjustment covariates				
Academic degree (vs. BSc/technician)				
PharmD	-0.086 (0.021)	-4.05	(-0.128, -0.045)	<0.001
Age group (vs. 20-30 years)				
31-40 years	-0.015 (0.026)	-0.58	(-0.066, 0.036)	0.564
41+ years	-0.015 (0.049)	-0.31	(-0.111, 0.081)	0.758
Years in practice (vs. <5 years)				
5-10 years	0.037 (0.027)	1.36	(-0.017, 0.090)	0.175
10-15 years	0.019 (0.040)	0.48	(-0.060, 0.098)	0.631
15+ years	0.102 (0.041)	2.48	(0.021, 0.183)	0.013
Total patient/day	0.001 (0.001)	1.36	(-0.000, 0.002)	0.175

When isolating the "core use" domain (routine instructions), the analysis reveals a pattern driven primarily by internal attributes and workflow intensity (Table 7). The habit of checking patient understanding remained a robust positive predictor (AME=0.103, p<0.001). Interestingly, workload pressure was positively associated with the provision of core instructions; pharmacists reporting high pressure scored 4.5 percentage points higher (AME=0.045, p=0.005) than those reporting no pressure. Conversely, lower self-efficacy significantly depressed performance; pharmacists rating their own counseling quality as "fair or lower" scored 10.7 percentage points lower (AME=-0.107, p<0.001) than their "excellent" counterparts. Consistent with the overall model, PharmD graduates scored significantly lower on this domain (AME=-0.047, p=0.004).

**Table 7:** Multivariable beta regression analysis of factors associated with the core use counseling Model pseudo R²=0.229; AIC=-660.1, log likelihood=342.1. AME, average marginal effect; SE, standard error; BSc, Bachelor of Science in Pharmacy; PharmD, Doctor of Pharmacy; AIC, Akaike information criterion

Predictor	AME (SE)	z-value	95% CI	p-value
Self-rated quality (vs. excellent)				
Good	-0.038 (0.015)	-2.57	(-0.067, -0.009)	0.010
Fair or lower	-0.107 (0.030)	-3.55	(-0.166, -0.048)	<0.001
Check understanding (vs. less frequent)				
Frequent	0.103 (0.027)	3.74	(0.049, 0.156)	<0.001
Barrier: workload pressure (vs. no)	0.045 (0.016)	2.82	(0.014, 0.076)	0.005
Pharmacy has assistant (vs. no)	0.019 (0.017)	1.13	(-0.014, 0.052)	0.258
Adjustment covariates				
Academic degree (vs. BSc/technician)				
PharmD	-0.047 (0.016)	-2.91	(-0.079, -0.015)	0.004
Age group (vs. 20-30 years)				
31-40 years	-0.033 (0.020)	-1.66	(-0.072, 0.006)	0.096
41+ years	-0.058 (0.043)	-1.36	(-0.141, 0.026)	0.175
Years in practice (vs. <5 years)				
5-10 years	0.043 (0.021)	2.03	(0.002, 0.085)	0.042
10-15 years	0.031 (0.031)	1.00	(-0.030, 0.091)	0.320
15+ years	0.084 (0.030)	2.78	(0.025, 0.143)	0.006
Total patient/day	0.000 (0.000)	1.03	(-0.000, 0.001)	0.305

The determinants of "risk communication" differed fundamentally from the core domain, showing a unique sensitivity to structural capacity (Table 8). The presence of a pharmacy assistant was the primary positive predictor, associated with a 9.9 percentage point increase (AME=0.099, p=0.010) in the provision of safety counseling. Unlike the core use model, risk communication was not significantly associated with academic degree (p=0.111) or workload pressure (p=0.349). However, self-perception remained a relevant factor; pharmacists who rated themselves merely as "good" (vs. "excellent") showed an 11.0 percentage point decline (AME=-0.110, p=0.003) in risk scores.

**Table 8:** Multivariable beta regression analysis of factors associated with the risk communication counseling Model pseudo R²=0.108; AIC=-189.4, log likelihood=106.7. AME, average marginal effect; SE, standard error; BSc, Bachelor of Science in Pharmacy; PharmD, Doctor of Pharmacy; AIC, Akaike information criterion

Predictor	AME (SE)	z-value	95% CI	p-value
Self-rated quality (vs. excellent)				
Good	-0.110 (0.037)	-3.03	(-0.182, -0.039)	0.003
Fair or lower	-0.061 (0.055)	-1.12	(-0.168, 0.046)	0.264
Check understanding (vs. less frequent)				
Frequent	0.068 (0.049)	1.39	(-0.028, 0.164)	0.165
Barrier: workload pressure (vs. no)	-0.033 (0.035)	-0.94	(-0.103, 0.036)	0.349
Pharmacy has assistant (vs. no)	0.099 (0.039)	2.57	(0.023, 0.175)	0.010
Adjustment covariates				
Academic degree (vs. BSc/technician)				
PharmD	-0.058 (0.037)	-1.59	(-0.130, 0.013)	0.111
Age group (vs. 20-30 years)				
31-40 years	0.019 (0.045)	0.41	(-0.070, 0.106)	0.681
41+ years	0.097 (0.073)	1.33	(-0.046, 0.239)	0.184
Years in practice (vs. <5 years)				
5-10 years	-0.002 (0.045)	-0.05	(-0.091, 0.087)	0.964
10-15 years	0.034 (0.064)	0.54	(-0.091, 0.160)	0.591
15+ years	0.014 (0.087)	0.16	(-0.157, 0.184)	0.875
Total patient/day	0.001 (0.001)	1.08	(-0.001, 0.003)	0.278

**Figure 2:**
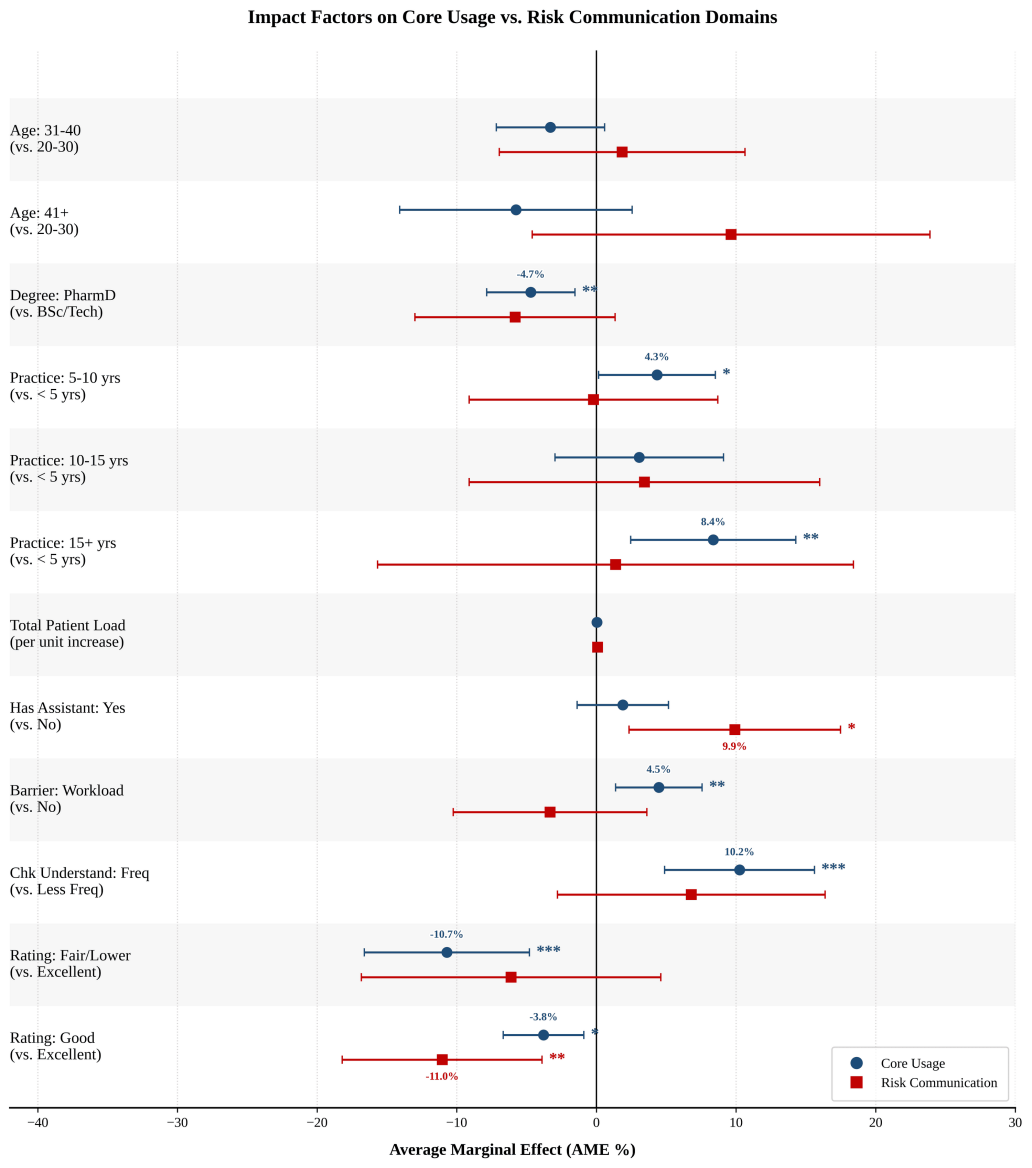
Forest plot of AME on counseling domains Markers represent the estimated percentage-point change in counseling scores associated with each predictor, with blue circles corresponding to the core use domain and red squares corresponding to the risk communication domain. Horizontal error bars indicate 95% confidence intervals. The vertical line at 0 represents no effect; markers to the right indicate a positive association, while markers to the left indicate a negative association. Asterisks denote statistical significance levels (*p<0.05, **p<0.01, ***p<0.001). AME, average marginal effects; PharmD, Doctor of Pharmacy

Figure 2 provides a comparative visualization of these effects, illustrating the functional divergence between the two domains. The forest plot reveals a clear decoupling of drivers: while "workload pressure" shifts performance positively for core use (blue markers), it fails to engage risk communication (red markers), which remain non-significant. In contrast, the "has assistant" variable displays the inverse pattern, serving as a distinct structural enabler for safety counseling (shifting the red marker significantly to the right) while showing negligible impact on routine instructions.

## Discussion

The findings of this study provide a granular view of community pharmacy practice in the Jazan region, revealing a distinct behavioral dichotomy. While pharmacists demonstrated high fidelity to routine technical instructions, there was a marked reduction in the provision of safety-critical risk information. This pattern of selective prioritization appears to be driven by two fundamentally different mechanisms: core use counseling is associated with internal professional habits and self-efficacy, whereas risk communication is uniquely sensitive to the structural capacity of the practice environment, specifically the presence of support staff. These results suggest that in a high-volume community setting, the decision to counsel on safety risks is less a function of knowledge and more a function of operational bandwidth.

The observed gap between the frequent provision of dosage instructions and the infrequent discussion of side effects aligns with previous descriptive work in Saudi Arabia [10,23,24] and reflects a broader global trend where pharmacists prioritize directive counseling over complex safety dialogue [13,25]. This may be interpreted as a form of clinical triage. In a busy retail environment, confirming the dose and route is a rapid, binary verification that fits within the workflow. In contrast, explaining potential adverse effects involves a nuanced negotiation of benefit versus harm, which Ferner and Aronson (2006) describe as cognitively demanding and time-intensive [14]. Furthermore, qualitative evidence suggests that pharmacists may actively filter risk information to avoid causing patient alarm or non-adherence [26]. Our data implies that without specific enablers, this "high-friction" safety counseling is the first activity to be shed under pressure.

The most significant enabler identified in this study was the presence of a pharmacy assistant, which was the sole positive predictor for risk communication scores. This aligns with work sampling studies by Walker et al. (2020), which demonstrated that the delegation of technical tasks to support staff directly correlates with increased pharmacist time for clinical services [18]. In the context of Jazan, where 60% of pharmacists cited workload pressure as a major barrier, assistants likely function as operational stabilizers. By managing logistical duties such as inventory and billing, they may reduce the cognitive clutter of the dispensing process, thereby preserving the pharmacist's capacity to engage in the more complex dialogue required for risk assessment. This supports the argument by Frost and Adams (2017) that effective task delegation is a prerequisite for the consistent delivery of advanced cognitive services [19].

Interestingly, while workload pressure was a barrier to counseling in general, the regression model showed a positive association between pressure and core use scores. This seemingly paradoxical finding might reflect the routinization of high-volume practice. In busy pharmacies, the workflow often demands strict adherence to standard operating procedures, where repeating the dose and directions becomes a drilled efficiency [17]. However, this efficiency did not extend to risk communication, which showed no improvement with pressure. This reinforces the hypothesis that while pressure can enforce the basics, it suppresses the expansion into safety counseling unless structural support is available.

For routine instructions, the driver appears to be the pharmacist's own professional engagement. The strong association between checking patient understanding and higher core scores suggests that counseling is an interactive loop; the act of verifying comprehension naturally compels the pharmacist to provide more detailed instructions. This is consistent with findings linking self-efficacy and professional commitment to higher performance in routine tasks [15,16]. Conversely, the significant drop in scores among those with lower self-rated quality indicates that professional confidence is a rate-limiting step. If a pharmacist does not perceive their service as high-quality, they are statistically less likely to perform even the basic counseling functions.

The results regarding academic degrees offer a critical insight into the interaction between education and environment. The finding that PharmD holders did not outperform, and in some metrics scored lower than, their BSc colleagues challenges the assumption that advanced clinical degrees automatically translate into better practice behaviors in all settings. This phenomenon has been observed in other contexts, where the demands of the retail environment were found to override the educational background of the practitioner [27,28]. It is possible that in the absence of a supportive infrastructure (such as private counseling areas or sufficient staffing), the advanced clinical skills of PharmD graduates remain latent. This suggests a systemic mismatch where the workforce is over-qualified for the logistical role they are often forced to play, potentially leading to role dissonance that impacts performance.

Limitations

These findings should be interpreted in light of several limitations. First, the data relies on self-reported frequencies, which may be subject to social desirability bias, although the variation in scores suggests participants were discriminating in their responses. Second, the cross-sectional design prevents causal inference; we can associate assistants with higher scores, but we cannot prove they caused the improvement. Additionally, the "risk communication" domain was assessed using a two-item scale (side effects and contraindications). While mathematically reliable, we acknowledge that this may not encompass all dimensions of comprehensive safety counseling, such as explicitly screening for drug-drug interactions. However, this focus aligns with the nature of at-counter POS interactions, which are designed for brief clinical triage rather than extensive medication therapy management sessions, where severe interactions are often conceptually communicated as contraindications. Finally, the study was conducted in the Jazan region, and while the workforce structure is similar to other regions of Saudi Arabia, local practice cultures may influence generalizability.

Future prospective 

As this study relied on cross-sectional data, future research would benefit from longitudinal designs to observe how counseling patterns evolve as staffing models or workload pressures fluctuate over time. Additionally, qualitative inquiry could provide deeper insight into why PharmD graduates in this specific setting do not outperform their peers, exploring whether this stems from role ambiguity or workflow constraints. Finally, while we identified predictors of counseling frequency, further studies are needed to determine if increased counseling on risk topics directly correlates with improved patient outcomes, such as medication adherence or error reduction, in the Saudi context.

## Conclusions

This study highlights a distinct pattern in community pharmacy practice where routine technical instructions are prioritized over complex safety counseling. The findings suggest that while internal professional factors drive standard interactions, the capacity to engage in risk communication is significantly associated with the presence of support staff. This indicates that the gap in safety counseling may be less about pharmacist knowledge and more related to the operational environment. Therefore, efforts to enhance patient safety should consider not only the clinical training of pharmacists but also the structural support available to them, recognizing that adequate workforce assistance may be a relevant factor in facilitating comprehensive patient care.

## Appendices

 Appendix 1

**Table 9:** Community pharmacist data collection instrument The counseling behavior items (questions seven-14) were developed by the authors based on the medication counseling content guidelines outlined by the FIP [4]. DRP, drug-related problem; FIP, International Pharmaceutical Federation

Domain	Item (question)	Response options/scale
I. Demographics	1. Age	☐ 20-30 years old ☐ 31-40 years old ☐ 41-50 years old ☐ >50 years old
	2. Academic degree	☐ BSc ☐ PharmD ☐ Technician pharmacist
	3. Years in practice	☐ <5 years ☐ 5-10 years ☐ 10-15 years ☐ 15+ years
II. Practice environment	4. Do you have an assistant employee in the pharmacy?	☐ Yes ☐ No
	5. Approximate number of patients per day with a prescription:	☐ 0-10 ☐ 11-20 ☐ 21-30 ☐ 31-40 ☐ >40
	6. Approximate number of patients per day without a prescription:	☐ 0-10 ☐ 11-20 ☐ 21-30 ☐ 31-40 ☐ >40
III. Counseling practices (CCI-8)	Stem: "How often do you inform patients about the following when dispensing a medication?"	Scale: 1 = never; 2 = sometimes; 3 = often; 4 = usually; 5 = always
	7. Indications	Use 5-point scale
	8. Route of administration	Use 5-point scale
	9. Dose	Use 5-point scale
	10. Directions for use	Use 5-point scale
	11. Duration of therapy	Use 5-point scale
	12. Special directions	Use 5-point scale
	13. Possible side effects	Use 5-point scale
	14. Contraindications	Use 5-point scale
IV. Perceptions & behaviors	15. How often do you make sure that patients understand their medications?	5-point scale: (never, sometimes, often, usually, always)
	16. How would you rate the quality of counseling services currently provided by community pharmacists?	☐ Excellent ☐ Good ☐ Fair ☐ Poor ☐ Needs improvement
	17. To what extent do you agree that pharmacists can reduce DRPs by providing consultations?	6-point scale: (strongly disagree, disagree, slightly disagree, slightly agree, agree, strongly agree)
	18. How knowledgeable do you believe community pharmacists are for managing DRPs in polypharmacy patients?	☐ Very knowledgeable ☐ Knowledgeable ☐ Moderately knowledgeable ☐ Unknowledgeable
V. Barriers	19. What do you think is the biggest barrier to providing sufficient counseling? (select all that apply)	☐ Lack of time ☐ Workload pressure ☐ Lack of patient acceptance ☐ Lack of financial support ☐ No patient medical history ☐ Lack of trust in pharmacist
